# Assessing genetic and phenotypic diversity to advance multi-trait selection in *Cyclocybe chaxingu*

**DOI:** 10.3897/imafungus.17.194629

**Published:** 2026-09-03

**Authors:** Jinlong Yi, Ge Su, Qiang Yang, Haiyang Song, Jianping Xu, Minghui Chen, Jianping Zhou, Hua Yin, Yusufjon Gafforov, Dianming Hu

**Affiliations:** 1 Bioengineering and Technological Research Center for Edible and Medicinal Fungi, Jiangxi Agricultural University, Nanchang, China Bioengineering and Technological Research Center for Edible and Medicinal Fungi, Jiangxi Agricultural University Nanchang China; 2 Nanchang Key Laboratory of Edible and Medicinal Fungi, Jiangxi Agricultural University, Nanchang, China Nanchang Key Laboratory of Edible and Medicinal Fungi, Jiangxi Agricultural University Nanchang China; 3 Jiangxi Provincial Key Laboratory of Excavation and Utilization of Agricultural Microorganisms, Jiangxi Agricultural University, Nanchang, China Jiangxi Provincial Key Laboratory of Excavation and Utilization of Agricultural Microorganisms, Jiangxi Agricultural University Nanchang China; 4 Department of Biology, McMaster University, 1280 Main St West, Hamilton, Ontario, L8S 4K1, Canada Department of Biology, McMaster University Hamilton Canada; 5 Department of Botany and Genetics, Faculty of Biology and Ecology, National University of Uzbekistan, Tashkent 100174, Uzbekistan Department of Botany and Genetics, Faculty of Biology and Ecology, National University of Uzbekistan Tashkent Uzbekistan; 6 Department of Medicine, Faculty of Medicine, Alfraganus University, Tashkent, 100190, Uzbekistan Department of Medicine, Faculty of Medicine, Alfraganus University Tashkent Uzbekistan; 7 Faculty of Chemical Technology, Fergana State Technical University, Fergana 150100, Uzbekistan Faculty of Chemical Technology, Fergana State Technical University Fergana Uzbekistan

**Keywords:** Barcoding, breeding, diversity, edible mushroom, germplasm evaluation, heritability, multivariate analysis, selection index

## Abstract

*Cyclocybe chaxingu* is a major cultivated edible mushroom in China; however, the relationship between its genetic diversity and agronomic performance remains insufficiently characterised. In this study, 100 strains were sampled, sequenced at two gene fragments (LSU and TEF1-α) and analysed for strain relationships at these loci. Ten agronomic traits were quantified under standardised cultivation conditions. Significant differences amongst strains were observed for all traits (ANOVA, *p* < 0.01). Amongst the ten traits, the greatest variation was found in the total number of fruiting bodies (CV 45.6%), followed by average fruiting-body weight (CV 35.0%). Principal component analysis accounted for 83.98% of the variance across three axes: PC1 (57.74%) represented a size and mass gradient (cap thickness, stipe diameter, cap diameter, gill width, average fruiting-body weight versus fruiting-body number), PC2 (15.87%) reflected stipe elongation and PC3 (10.37%) captured the timing of first harvest and earliest yield. Hierarchical clustering identified four phenotypic branches and five operational colour groups, each displaying strong differences across traits. Phylogenetic analysis at the two loci grouped strains into four distinct clusters, which partially overlapped with phenotype-based clusters. Yield was positively correlated with size-related traits and gill width and negatively correlated with fruiting-body number. Time to first harvest and earliest yield were associated with cap thickness and gill width. Stipe diameter, stipe length and gill width are proposed as practical DUS indicator traits for breeding, with size traits serving as complementary selection targets, thereby providing a quantitative breeding framework for *C. chaxingu*.

## Introduction

*Cyclocybe chaxingu* (N.L. Huang) Q.M. Liu, Y. Gao & D.M. Hu, a member of the basidiomycete family *Hemipholiotaceae*, is one of the major cultivated edible mushrooms in China (Xu et al. 2006; Wang 2013; Liu 2020; Niskanen et al. 2026). Its fruiting bodies have high nutritional value, distinctive aroma and abundant bioactive compounds (Xu et al. 2006). First described from tea-tree forests in Jiangxi (Wang 2013), *C. chaxingu* has also been used in traditional medicine to regulate liver and spleen function, improve vision and mitigate digestive ailments. Polysaccharides from this and other medicinal mushrooms, often termed “biological response modifiers”, have shown immunostimulatory, anti-tumour, antiviral, antioxidant, hepatoprotective, hypoglycaemic and related activities with minimal toxicity (Li 1985; Zhou et al. 1989, 1995; Fang et al. 1996; Zhou and Meng 1997; Cheng and Zhu 1999; Wang and Guan 2000; Huang et al. 2001; Zhou 2001; Li et al. 2002; Sun et al. 2003; Qin et al. 2004). With successful domestication and readily available cultivation substrates, *C. chaxingu* has become an important crop in several Chinese provinces, contributing to rural economies (Lu and Gao 2005; Ruan and Dai 2009; Chen et al. 2012a).

Taxonomically, *C. chaxingu* belongs to the *C. cylindracea* complex that also includes *C. aegerita* and related species (Wang et al. 1998; Cai et al. 2007, 2011; Jin 2013; Wang et al. 2021). Although these mushrooms are sometimes collectively marketed as *C. cylindracea* (syn. *Agrocybe cylindracea*), multi-locus phylogenetic analyses using ITS, LSU and RPB2 gene sequences confirmed that *C. chaxingu* is a distinct species (Chen et al. 2012b; Błaszkowski et al. 2015; Liu et al. 2021; Yang et al. 2025). Accurate species delimitation underpins germplasm evaluation and breeding. As shown for other edible fungi, such as *Lentinula edodes*, *Pleurotus giganteus* and *Flammulina velutipes*, integrating phenotypic and molecular data can provide robust foundations for strain improvement (Gong et al. 2014; Wang et al. 2018; Zhang et al. 2022; Gu et al. 2024; Yan et al. 2024). Indeed, research on *Agrocybe aegerita*, a close relative of *C. chaxingu* within the same *C. cylindracea* complex, revealed considerable genetic variation in agronomic traits, underscoring the value of diversity assessments for strain and trait selection and breeding (Wang et al. 2012, 2022, 2024; Zhang et al. 2019).

Despite its nutritional and economic importance, progress in *C. chaxingu* has been constrained by insufficient characterisation of germplasm resources, underdeveloped breeding systems and the absence of Distinctness, Uniformity and Stability (DUS) test guidelines (Sonnenberg et al. 2017; Dong et al. 2022). To address these gaps, we provide, to our knowledge, the first large-scale assessment of agronomic and genetic diversity in *C. chaxingu*, analysing 100 strains with correlation and clustering statistics of agronomic traits alongside molecular data. The findings establish a foundation for germplasm collection, selection of superior varieties and the development of DUS guidelines for this economically important edible mushroom.

## Materials and methods

### Strains

One hundred strains of *Cyclocybe chaxingu* were collected and are maintained at the Jiangxi Agricultural University Culture Collection Center (JAUCC). These strains came from three different sources: private mushroom farms, culture collections and the wild. Geographically, these 100 strains came from 12 provinces and two municipalities in China. These strains were selected for this study to represent the broad geographic and ecological origins of this species distributions. The details of these strains are presented in Suppl. material 1.

### Strain cultivation and fruiting

Our mushroom fruiting protocol followed that described in (Gao et al. 2026). Briefly, to fruit these strains, their mycelia were first grown on PDA (per litre: potato infusion from 200 g potato, 20 g glucose, 20 g agar; natural pH) and incubated at 25 °C in the dark for 7 d. Fully colonised agar blocks were then transferred to sterilised solid substrate packed in polypropylene filter bags. The cultivation substrate (% w/w, wet basis) consisted of 15% cottonseed hulls, 20% coarse sawdust, 20% fine sawdust, 20% bagasse, 15% wheat bran, 5% soybean meal, 3% cornmeal, 1% gypsum and 1% lime, adjusted to ~ 65% moisture. During incubation, environmental conditions were 20–25 °C, 60–70% RH, with adequate fresh-air exchange and darkness. After ~ 50 d, when bags were fully colonised, they were opened to induce primordia and fruiting at 22 °C, ~ 95% RH and ~ 600 lx diffuse light.

### DNA extraction and amplification

To analyse strain genotypes, their mycelia were ground in liquid nitrogen and genomic DNA from each strain was extracted by the CTAB method (Porebski et al. 1997). Universal fungal primer pairs were used to amplify regions of the large subunit of the ribosomal RNA gene (LSU) and the translation elongation factor 1-α (TEF1-α). These two markers were chosen for this study for several reasons. The first was their readily available universal primers that could be used to obtain sequences from all of our strains. The second was that these two gene fragments have been used for fungal phylogenetics and the identification of putative cryptic species, including as secondary fungal DNA barcodes (Xu 2016). The third is that, at least for TEF1- α, intraspecific variations have been reported within many fungal species (Buyck and Hofstetter 2011). PCR products were verified by 1% agarose gel electrophoresis and Sanger sequenced by Sangon Biotech (Shanghai) Co., Ltd. (Yang and Qin 2023). Primers and cycling profiles are listed in Table 1.

**Table 1.** Primers and PCR programmes.

**Locus**	**Primer**	**Sequence (5’→3’)**	**PCR amplification protocol**
LSU	LR0R	ACCCGCTGAACTTAAGC	94 °C 3 min; 35 cycles of 94 °C 30 s, 55 °C 50 s, 72 °C 1 min; 72 °C 10 min
	LR5	TCCTGAGGGAAACTTCG	
TEF1-α	TEF1-983F	GCYCCYGGHCAYCGTGAYTTYAT	96 °C 2 min; 40 cycles of 96 °C 45 s, 52 °C 30 s, 72 °C 90 s; 72 °C 5 min
	TEF1-1567R	ACHGTRCCRATACCACCRATCTT	

### Sequence curation, alignment and phylogeny

Raw reads were inspected and assembled to consensus chromatograms; sequences were queried by NCBI BLAST for preliminary identification. Locus-wise alignments were generated with MAFFT (default/normal mode) (Katoh and Standley 2013), trimmed and concatenated in MEGA (Sudhir et al. 2018). Phylogenetic analyses were run in PhyloSuite (Zhang et al. 2020) with ModelFinder to select the best-fit partition models (Kalyaanamoorthy et al. 2017). MrBayes v.3.2.7a was used under a partitioned scheme (two parallel runs, 2,000,000 generations, sampling every 1,000; first 25% discarded as burn-in). Convergence was checked by the average standard deviation of split frequencies (< 0.01)

### Agronomic trait assessment

**Figure 1.**
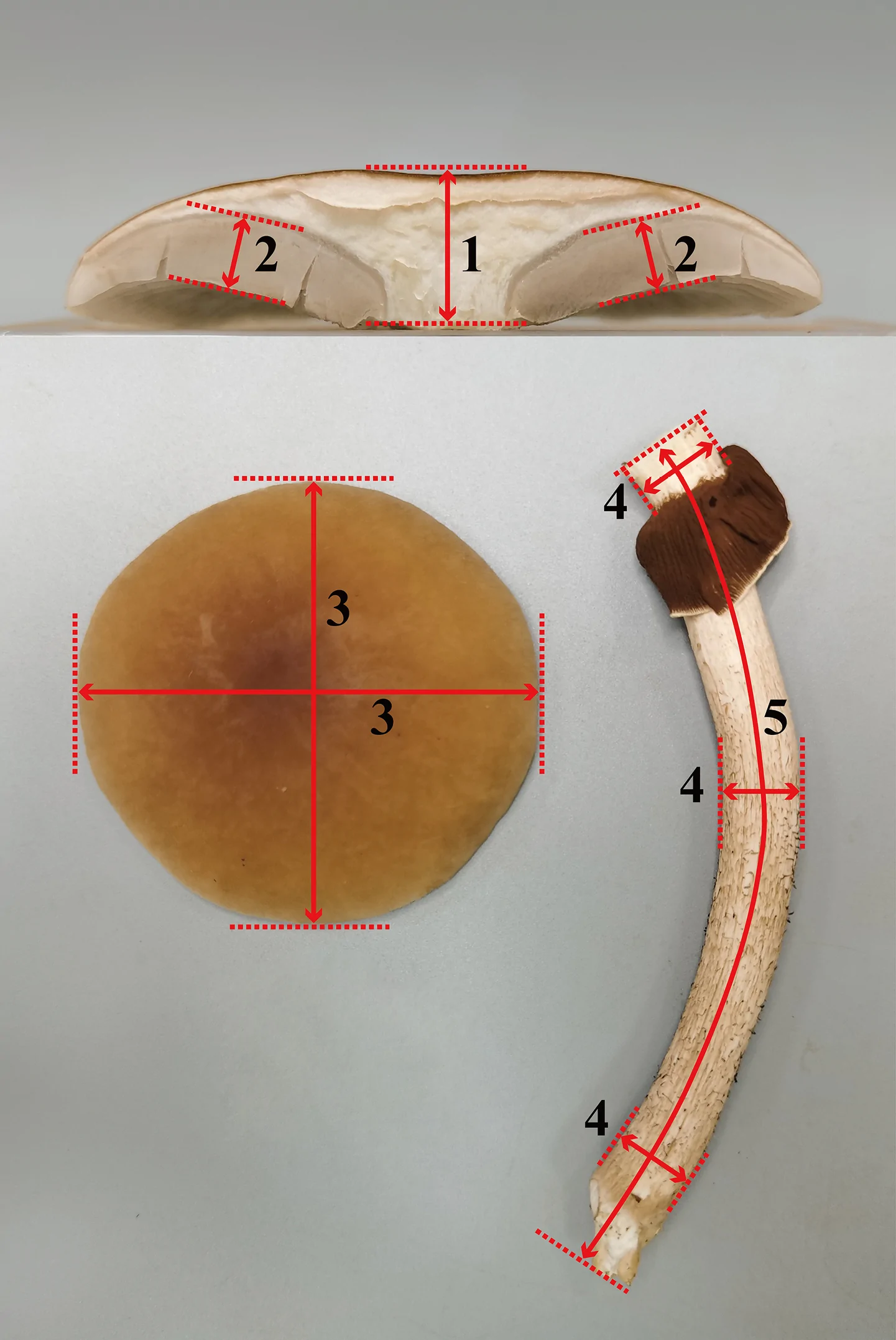
Measurement scheme for fruiting-body traits: (1) Cap thickness; (2) Gill width; (3) Cap diameter; (4) Stipe diameter; (5) Stipe length.

Ten agronomic traits were quantified on market-mature fruiting bodies (definitions in Table 2; measurement scheme in Fig. 1). Specifically, for each strain, six bags were prepared and inoculated with agar plugs of the same dimensions. All bags were cultivated under identical conditions for 55 days, after which they were opened and randomly arranged to initiate fruiting. Data for DHF, YLD, AFW and FBN were collected individually for each bag. For traits SL, SD, CD, CT and GW, ten representative fruiting bodies with standard shape and a range of sizes were chosen from each bag and measured (Table 2). Subsequently, the mean, standard deviation and other essential statistics were computed for every trait, as described below.

**Table 2.** Quantitative agronomic traits and measurement definitions for *Cyclocybe chaxingu*.

**Trait (abbrev.)**	**Abbreviated Unit**	**Definition / Measurement**
Days to first harvest (DFH)	d	Time from mycelium inoculation to the first harvestable fruiting bodies.
Total yield (YLD)	g·bag^-1^	Total fresh mass of all fruiting bodies harvested per bag over the fruiting period (all flushes) (in grams).
Average fruiting body weight (AFW)	g	Mean fresh mass per fruiting body (in grams).
Total number of fruiting bodies (FBN)	count·bag^-1^	Cumulative count of fruiting bodies harvested per bag over the fruiting period.
Stipe length (SL)	mm	Distance from the pileus attachment to the stipe base at the substrate surface (in millimetres).
Stipe diameter (SD)	mm	Mean of diameters measured at the upper, middle and lower thirds of the stipe (in millimetres).
Cap diameter (CD)	mm	Mean of the maximum longitudinal and transverse pileus diameters (in millimetres).
Cap thickness (CT)	mm	Vertical distance from the pileus surface to the stipe–pileus junction (in millimetres).
Gill width (GW)	mm	Vertical distance at mid-gill from the lower edge to the upper margin (in millimetres).
Morphological index (MI)	—	Ratio CD / SL (dimensionless).

### Data analysis

All analyses were performed in R. Descriptive statistics (means, standard deviations, distributions) were computed with dplyr. Pairwise associations amongst the ten agronomic traits were estimated using Pearson’s r and visualised as correlation heatmaps with corrplot and pheatmap (Taiyun and Viliam 2021). For all analyses that involved multiple comparisons — specifically those performed with the corrplot, pheatmap, dendextend, FactoMineR and factoextra packages — P-values were adjusted to minimise false discovery rate (FDR).

Before multivariate analyses, trait values were z-standardised. Phenotypic dissimilarity between strains was defined as 1 – r (Pearson correlation across traits). Strains were grouped by hierarchical clustering (UPGMA); dendrogram construction and visualisation were performed with dendextend (Galili 2015) and UPGMA routines (Moulton et al. 2018). The resulting tree depicts phenotypic similarity patterns amongst the 100 strains.

Global structure was summarised using principal component analysis (PCA) with FactoMineR and the scores and loadings were visualised with factoextra (Iezzoni and Pritts 1991; Lê et al. 2008; Kassambara and Mundt 2016), providing a compact view of trait covariation and strain grouping in *C. chaxingu*.

## Results

### Descriptive statistics of agronomic traits

All strains produced fruiting bodies under the standardised cultivation conditions and ten traits were recorded for each of the 100 strains (Table 3). All 10 traits showed significant variation amongst strains (one-way ANOVA, p < 0.01). Coefficients of variation (CV) were highest for FBN (45.6%), followed by AFW (35.0%), MI (22.7%), GW (19.7%) and CT (19.7%), indicating pronounced phenotypic diversity in fruiting body number- and size-related traits within our strain collection. Moderate variations were also observed for the remaining five traits: SD (CV = 16.8%), YLD (14.6%), CD (14.2%) and SL (13.1%), while DFH varied the least (7.6%). Collectively, these metrics reveal substantial phenotypic diversity within *C. chaxingu*, providing abundant germplasm for downstream strain selection and breeding.

**Table 3.** Descriptive statistics of ten agronomic traits in *Cyclocybe chaxingu* (n = 100 strains).

**No**.	**Trait**	**Unit**	**Mean**	**Min**	**Max**	**SD**	**CV (%)**
1	DFH	d	73.23	61.33	93.00	5.58	7.62
2	YLD	g·bag^-1^	147.28	101.14	211.15	21.44	14.56
3	AFW	g	8.97	4.98	18.22	3.14	34.99
4	FBN	count·bag^-1^	31.70	8.75	81.82	14.46	45.60
5	SL	mm	67.55	44.04	86.68	8.85	13.09
6	SD	mm	7.61	5.09	12.23	1.28	16.83
7	CD	mm	43.16	32.57	61.52	6.14	14.23
8	CT	mm	9.45	6.36	18.10	2.24	19.72
9	GW	mm	3.83	2.68	5.88	0.76	19.72
10	MI	—	0.64	0.45	1.15	0.15	22.71

### Correlations amongst agronomic traits

**Figure 2.**
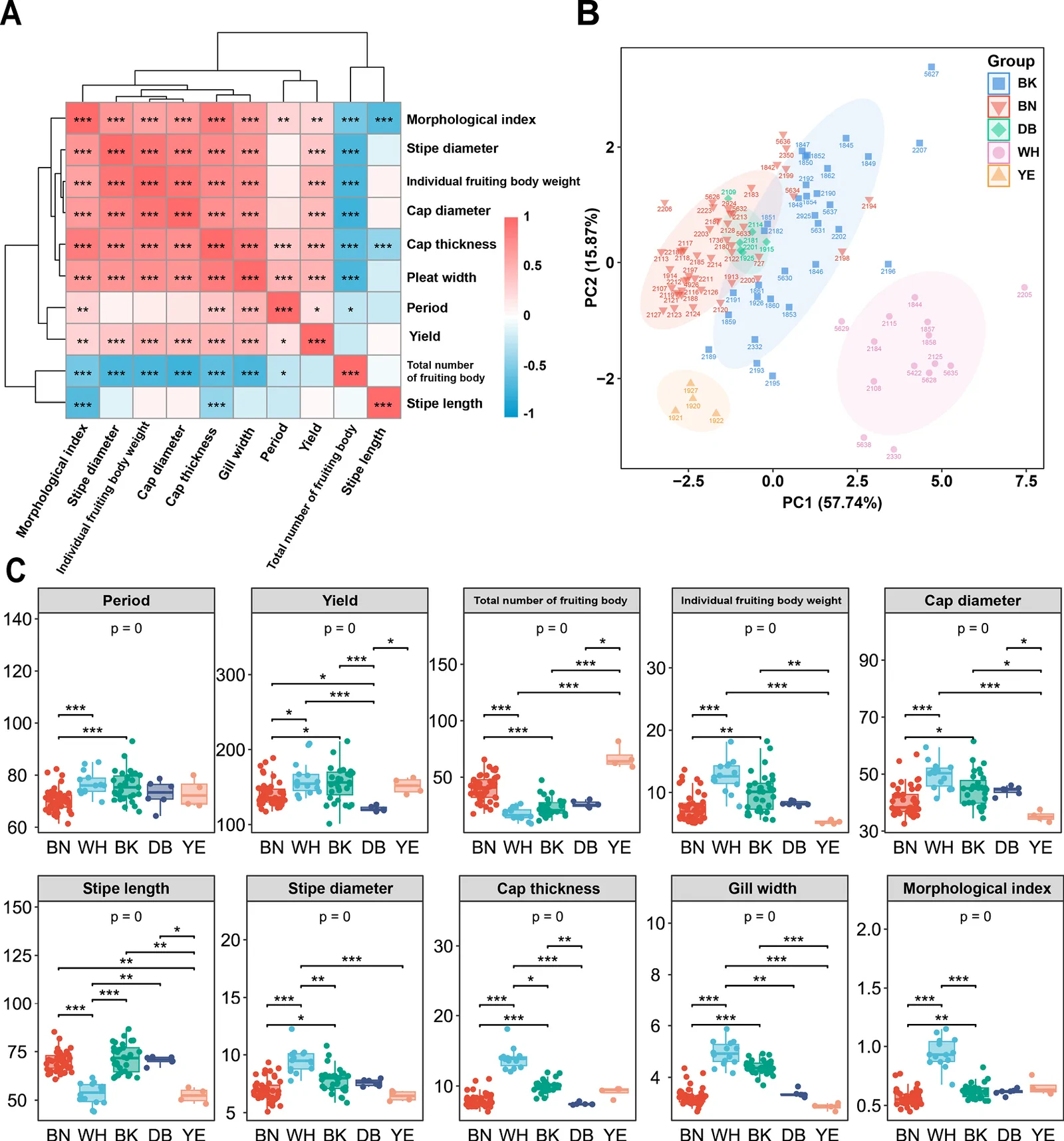
Multivariate patterns of agronomic traits in *Cyclocybe chaxingu*. **A** Pearson correlation matrix (hierarchically ordered). Asterisks denote significance levels (p < 0.05, p < 0.01, p < 0.001; false discovery rate (FDR)-adjusted); **B**PCA scores on PC1 (57.74%) and PC2 (15.87%). Ellipses indicate 95% normal-theory confidence regions for the five colour groups (BK, BN, DB, WH, YE); **C** Groupwise comparisons for ten traits. Boxes show IQR with median; whiskers = 1.5× IQR.

Correlation heatmaps (hierarchical ordering) revealed both positive and negative associations amongst traits (Fig. 2A). MI showed positive correlations with CD and CT and a negative correlation with SL, indicating that larger, thicker caps combined with shorter stipes yield higher MI values. SD correlated positively with AFW, CD and CT, but negatively with SL, suggesting that thicker stipes accompany heavier, larger fruiting bodies with shorter stipes. For yield-proximal patterns, YLD correlated positively with SD, AFW, CD, CT, GW (and weakly with DFH), but negatively with FBN, consistent with a trade-off between producing fewer, heavier versus many, lighter fruiting bodies. AFW was strongly and positively associated with SD, CD, CT and GW and negatively with FBN, reinforcing this pattern. The abbreviations in Fig. 2B and Fig. 2C are listed in Table 4.

**Table 4.** Practical recommendations for growers (field-orientated summary).

**Group**	**Typical phenotype (this study)**	**Selection/cultivation focus**
BK (Black)	Higher YLD; robust basidiomes; faster spore maturation	Target AFW, SD, CD, CT; maintain moderate FBN; manage ventilation to avoid hollow stipes
BN (Brown)	Shorter DFH (earlier harvest)	Select for earliness with acceptable AFW; pair with traits sustaining YLD (SD/CD)
DB (Dark brown)	Intermediate between BN and BK	Balance AFW and FBN; stabilise SL/SD for uniformity
WH (White)	Higher AFW; lower FBN	Emphasise size/mass traits (AFW, CD, CT, SD); monitor texture/whiteness for market quality
YE (Yellow)	Higher FBN, lower AFW/YLD	Thin clusters to reduce competition; select for AFW and SD/CD, while moderating FBN

Reporting note: In Fig. 2C, post-hoc contrasts are summarised as Tukey’s Honest Significant Difference (HSD) test letters (FDR-adjusted), which are shown above each box to replace multiple asterisk bars.

### Hierarchical clustering and phenotypic differentiation

**Figure 3.**
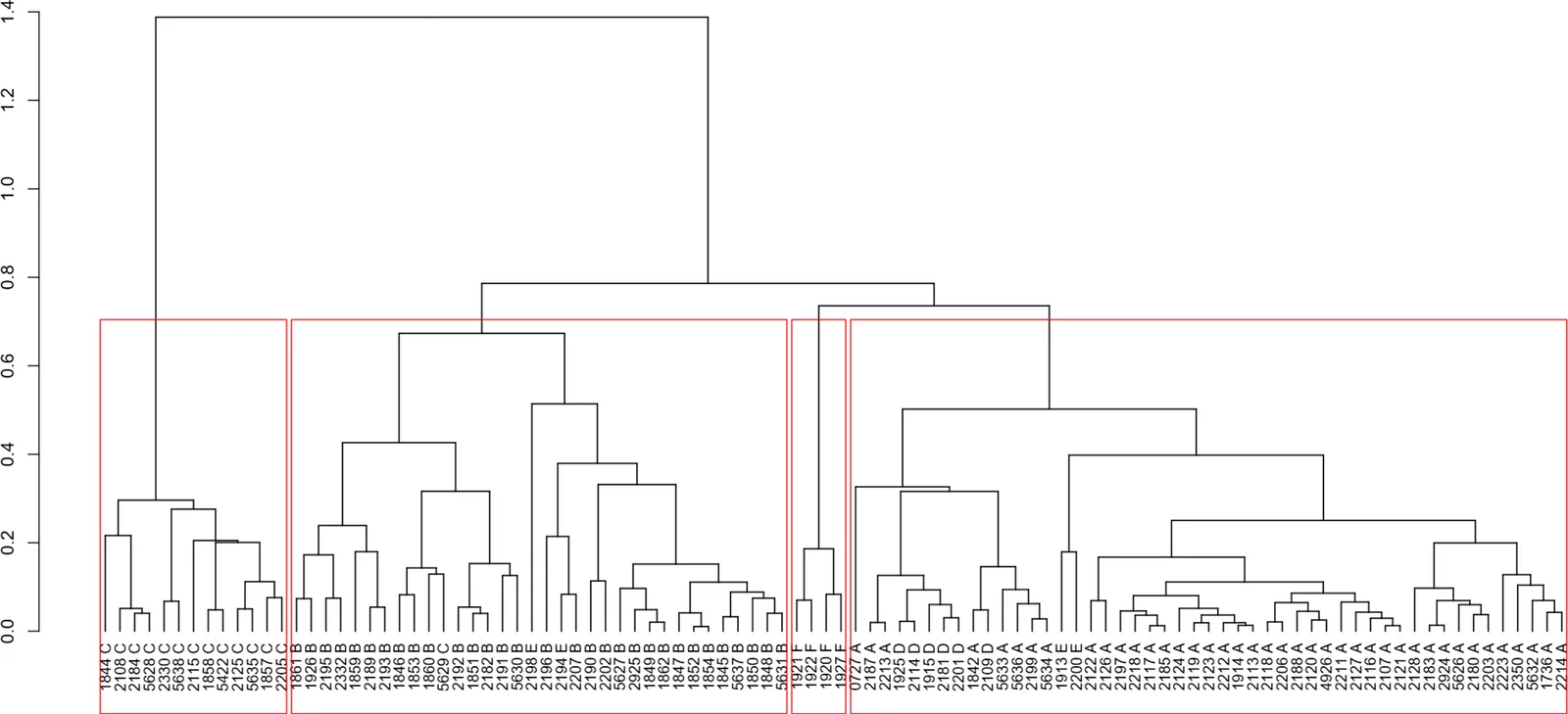
UPGMA dendrogram, based on ten traits of *Cyclocybe chaxingu*.

**Figure 4.**
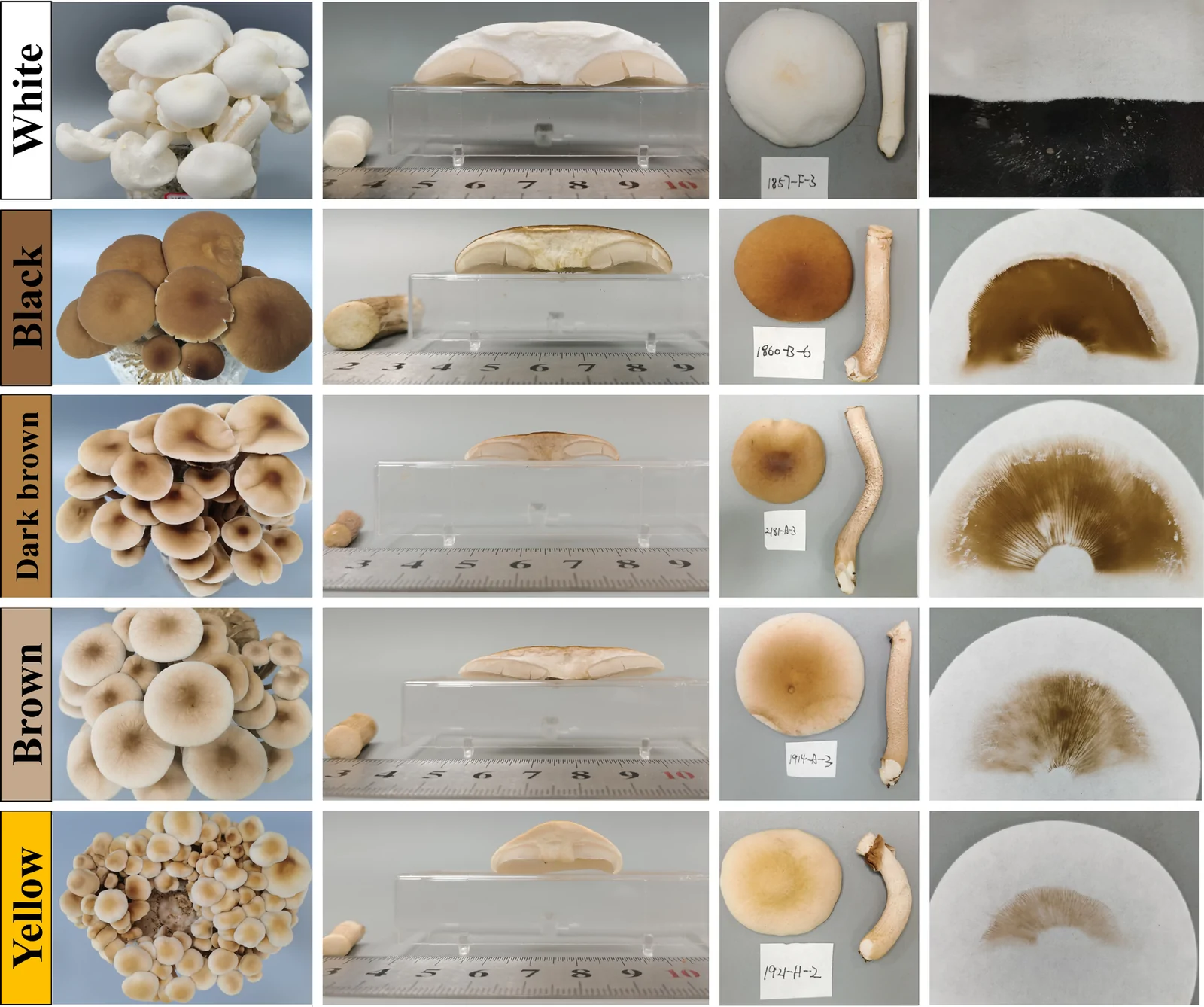
Representative morphology for the five fruiting body colour groups (WH, BK, DB, BN, YE), showing cluster form, longitudinal section, pileus/stipe view and spore print. Scale bars as indicated.

UPGMA clustering on standardised trait data grouped strains into four major phenotypic branches (Fig. 3). Basidiome colours largely tracked these branches and served as a practical discriminator; therefore, strains were partitioned into five colour-based groups: white (WH), black (BK), dark brown (DB), brown (BN) and yellow (YE) (Fig. 4). Between-group comparisons showed pervasive differences across traits (overall p < 0.001; Fig. 2C). Briefly, DFH was shortest in BN and longest in YE; YLD was highest in BK and lowest in YE; AFW tended to be higher in WH and lower in YE; FBN was highest in YE and lowest in WH. Significant differences were also observed in SL, SD, CD, CT, GW and MI, indicating multivariate differentiation amongst the five groups.

### Principal component analysis

The first three principal components (PCs) accounted for 83.98% of the total variance (PC1 = 57.74%, PC2 = 15.87%, PC3 = 10.37%; Table 5). PC1 loaded strongly on CT, AFW, SD, CD, GW, MI (positive) and FBN (negative), representing a size/mass axis contrasting a few large fruiting bodies with many small ones. PC2 was dominated by SL (positive) and, to a lesser extent, MI (negative), capturing a stipe-elongation gradient. PC3 loaded mainly on DFH (positive) and YLD (positive), describing earliness/yield timing. The PC1–PC2 scatter (Fig. 1B) showed partially separated clouds for the five colour groups: YE tended to the lower-left, WH to the lower-right, BN to the upper-left and BK near the upper-right; DB lay intermediate between BN and BK, suggesting that colour phenotypes are associated with multivariate agronomic patterns.

**Table 5.** Principal component loadings (on standardised traits) and variable contributions (%) for the first three PCs. [Values shown as loading (contribution %); use absolute loadings for interpretation. Variance explained: PC1 = 57.74%, PC2 = 15.87%, PC3 = 10.37%].

**Agronomic trait (abbrev.)**	**PC1 (57.74%)**	**PC2 (15.87%)**	**PC3 (10.37%)**
Days to first harvest (DFH)	0.336 (1.95)	–0.395 (9.84)	0.747 (53.81)
Total yield (YLD)	0.523 (4.73)	0.146 (1.34)	0.446 (19.19)
Total no. of fruiting bodies (FBN)	–0.772 (10.31)	–0.255 (4.09)	0.055 (0.29)
Average fruiting-body weight (AFW)	0.908 (14.29)	0.316 (6.28)	–0.113 (1.23)
Cap diameter (CD)	0.888 (13.67)	0.302 (5.74)	–0.082 (0.64)
Stipe length (SL)	–0.199 (0.69)	0.925 (53.93)	0.261 (6.59)
Stipe diameter (SD)	0.892 (13.78)	0.158 (1.58)	–0.255 (6.25)
Cap thickness (CT)	0.933 (15.07)	–0.243 (3.73)	0.000 (0.00)
Gill width (GW)	0.888 (13.65)	–0.051 (0.16)	0.248 (5.93)
Morphological index (MI = CD/SL) CD/SL)	0.827 (11.85)	–0.460 (13.30)	–0.251 (6.06)

### Phylogenetic evidence for intraspecific differentiation of *C. chaxingu*

**Figure 5.**
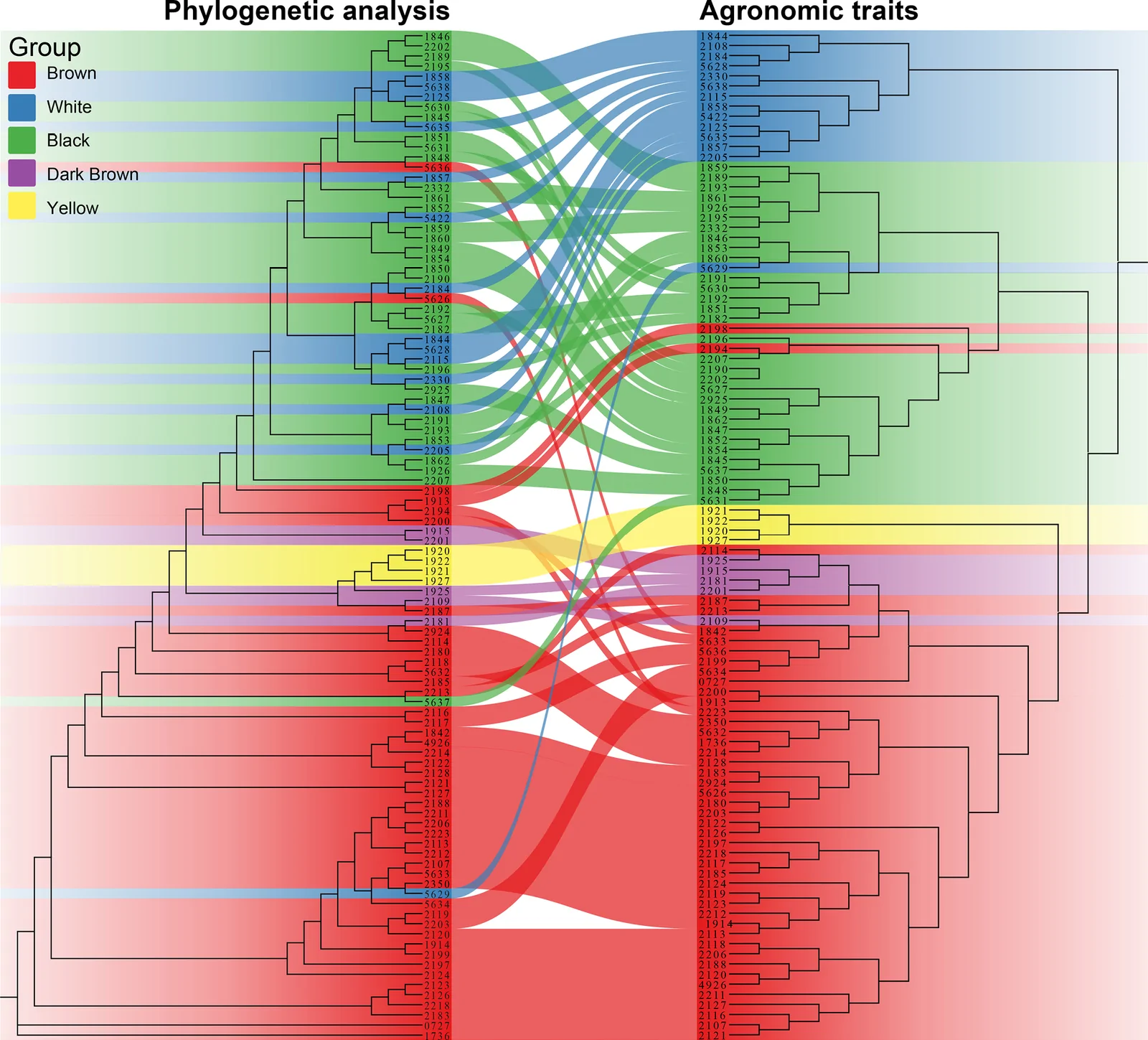
Correspondence between genetic and phenotypic clusters. Left: Bayesian phylogeny from concatenated LSU + TEF1-α sequences (support metrics as indicated). Right: UPGMA dendrogram from ten agronomic traits. Coloured alluvial links map strain membership between genetic clusters and phenotypic groups (WH, BK, DB, BN, YE).

To investigate the potential relationships between genetic and phenotypic differences amongst strains, we juxtaposed a Bayesian phylogeny from concatenated LSU + TEF1-α sequences with a phenotypic dendrogram derived from the ten agronomic traits (Fig. 5). The phylogeny resolved four principal genetic clusters, whereas phenotypic clustering defined five colour-coded groups (WH, BK, DB, BN, Y). WH and BK frequently co-clustered genetically and a subset of BN strains were mixed with DB/WH/BK. Overall, several phenotypic groupings were partially supported by the phylogeny, suggesting that some agronomic patterns reflect underlying genetic structure, while others may arise from phenotypic plasticity or polygenic variation beyond the resolution, based on the two sequenced loci.

## Discussion

### DUS implications for *Cyclocybe chaxingu*

Trait standardisation and DUS (Distinctness, Uniformity, Stability) guidelines are essential for variety protection and orderly industry growth. As fruiting and fruiting body features of edible mushrooms are highly sensitive to environmental conditions, DUS trials should minimsze environmental noise by testing candidate and reference varieties under identical conditions with adequate plot size and sample number to identify heritable variations; where feasible, use centralised test sites to improve accuracy and reproducibility (Chu et al. 2023). Under current Chinese practice, distinctness can be established when a test variety shows a repeatable, significant difference in ≥ 1 trait versus the reference. Based on our variance structure and between-strain contrasts, stipe diameter (SD), stipe length (SL) and gill width (GW) emerged as practical indicator traits for DUS testing in *C. chaxingu*.

### Phenotypic diversity and breeding value

Since the 1980s, *C. chaxingu* cultivation has expanded significantly in China (Liu et al. 2020; Gao et al. 2026). In our panel of 100 strains, all 10 traits showed significant variation amongst strains (ANOVA, p < 0.01), with the greatest trait value dispersion in FBN (CV 45.6%) and the second highest in AFW (CV 35.0%). This breadth of phenotypic diversity indicates rich germplasm diversity for selection and provides a foundation for breeding and variety protection (Li et al. 2024).

### Trait relationships and selection directions

Correlation patterns support two contrasting fruiting body yield strategies: (i) fewer, heavier fruiting bodies (higher AFW; larger CD/CT/SD) and (ii) many, lighter fruiting bodies (higher FBN). YLD correlated positively with AFW, SD, CD, CT, GW and negatively with FBN, suggesting that increasing cap/stipe size while moderating FBN is a plausible route to higher yield. DFH is associated with CT and GW, so these can serve as auxiliary indicators when selecting for shorter cycles. As correlations do not imply causation, these directions should be validated in multi-environment fruiting trials and through selection and response-to-selection trials.

Our analyses identified strains with superior performances in one to multiple economic traits. These strains are candidate parental strains for future breeding programmes that target the specific sets of traits for improvements. For example, we identified strains with either short cultivation periods, high overall productivity. These strains could serve as parental stocks for breeding new strains with three combined high-quality traits. In addition, SD, SL and GW may also be employed as practical metrics for *C. chaxingu* in DUS testing, offering valuable reference points for the outcomes of cultivation experiments conducted under diverse conditions.

### Group differentiation and multivariate structure

UPGMA clustering resolved four major phenotypic branches; inspection of basidiome colour supported five colour groups (WH, BK, DB, BN, YE) with pervasive differences across traits (overall *p* < 0.001). PCA indicated three interpretable axes: PC1 (57.74%) = size/mass (CT, AFW, SD, CD, GW vs. FBN); PC2 (15.87%) = stipe elongation (SL); PC3 (10.37%) = earliness/yield timing (DFH, YLD). Operationally, BK tended to have higher YLD, BN had shorter DFH and YE had many. but lighter fruiting bodies. Thus, fruiting body colour is a useful field cue, but because it can be influenced by environmental conditions, other quantitative trait descriptors should be included for more accurate identification of strain groupings.

### Genetic–phenotypic congruence

Concatenated LSU + TEF1-α sequences could intuitively reflect the five groups defined by phenotypic clustering, particularly groups BN, DB and YE. Despite overall genetic co-clustering between WH and BK, most admixed individuals clustered separately on distinct branches. This partial congruence suggests that some agronomic differentiation reflects genetic structure, while other patterns may arise from phenotypic plasticity or polygenic backgrounds not captured by the two sequenced loci. Future work with genetic crosses and denser markers (e.g. multilocus sequence typing, amplified fragment length polymorphisms and whole-genome sequencing) and formal congruence statistics (Mantel, Procrustes, adjusted Rand index) will refine these links.

### Limitations and future directions

Our study has several limitations. First, we analysed the fruiting pattern at only one site under a single cultivation condition. Multi-site, multi-fruiting condition and multi-season trials are needed to quantify G×E and trait repeatability (ICC). DUS panels will benefit from BLUP-based means and effect sizes (η^2^ or Cliff’s δ) and from the inclusion of biological efficiency (BE) and quality traits (texture, aroma) alongside yield. Second, only two loci were sequenced and both loci are highly conserved within a species. Analysing more loci will allow greater differentiations amongst strains, which may help identify potential linkages between genomic regions and phenotypic traits. The tetrapolar heterothallic system of *C. chaxingu* (Cai et al. 2007; Zheng et al. 2007) is ideal for investigating such relationships and can be harnessed in breeding schemes.

## Conclusions

*Cyclocybe chaxingu* demonstrates substantial phenotypic diversity along clear and interpretable axes, including size or mass, stipe elongation and earliness. SD, SL and GW are identified as primary DUS indicator traits, supported by multi-trait patterns, such as AFW, CD, CT and FBN, which provide information for selection. While colour-based grouping facilitates field operations, it should be complemented with quantitative descriptors. Practical recommendations for the five groups are presented in Table 4. These findings establish a practical framework for DUS characterisation, parental selection and breeding to enhance yield and reduce production cycles, while highlighting the importance of multi-environment evaluation and more comprehensive genotyping.

## Additional information

### Artificial Intelligence (AI) use

The authors accept full responsibility for the content of the manuscript, including the disclosure of any use of AI.

No AI tools were used in the preparation of this manuscript.

### Adherence to national and international regulations

All the fungal strains used in this study have been legally obtained, respecting the Convention on Biological Diversity (Rio Convention).

### Author ORCIDs

J. Yi https://orcid.org/0009-0009-0759-4888

G. Su https://orcid.org/0009-0009-9838-181X

Q. Yang https://orcid.org/0009-0003-7642-5080

H. Song https://orcid.org/0009-0001-4140-5720

J. Xu https://orcid.org/0000-0003-2915-2780

M. Chen https://orcid.org/0009-0006-3497-3312

J. Zhou https://orcid.org/0009-0003-2511-7686

H. Yin https://orcid.org/0000-0003-4611-8533

Y. Gafforov https://orcid.org/0000-0003-3076-4709

D. Hu https://orcid.org/0000-0002-4750-2871

### Data availability

All of the data that support the findings of this study are available in the main text or Supplementary Information.

## Supplementary materials

Supplementary material 1 Strain codes and their associated metadata Data type docx https://binary.pensoft.net/file/1763779This dataset is made available under the Open Database License (http://opendatacommons.org/licenses/odbl/1.0/). The Open Database License (ODbL) is a license agreement intended to allow users to freely share, modify, and use this Dataset while maintaining this same freedom for others, provided that the original source and author(s) are credited. Jin-Long Yi, Ge S, Qiang Yang, Hai-Yang Song, Ming-Hui Chen, Jian-Ping Zhou, Hua Yin, Dian-Ming Hu
