# Supplementary material for: Assessing genetic and phenotypic diversity to advance multi-trait selection in Cyclocybe chaxingu
Source: IMA Fungus. 2026 Sep 3;17:e194629. doi: 10.3897/imafungus.17.194629 (PMC13563116; doi:10.3897/imafungus.17.194629)
Supplement: Supplementary file 1 [file imafungus-17-e194629-s001.docx]

Table S1. The test strains.

| Strain Code | Source of Origin | GB numbers (LSU) | GB numbers (TEF1) |
| --- | --- | --- | --- |
| JAUCC0727 | Jiangxi Province | - | PX378091 |
| JAUCC1736 | Jiangxi Province | PX376348 | PX425488 |
| JAUCC1842 | Jiangxi Province | PX376349 | PX425489 |
| JAUCC1844 | Jiangxi Province | PX376350 | PX425490 |
| JAUCC1845 | Jiangxi Province | PX376351 | PX425491 |
| JAUCC1846 | Jiangxi Province | PX376352 | PX425492 |
| JAUCC1847 | Jiangxi Province | PX376353 | PX425493 |
| JAUCC1848 | Jiangxi Province | PX376354 | PX425494 |
| JAUCC1849 | Jiangxi Province | PX376355 | PX425495 |
| JAUCC1850 | Jiangxi Province | PX376356 | PX425496 |
| JAUCC1851 | Jiangxi Province | PX376357 | PX425497 |
| JAUCC1852 | Jiangxi Province | PX376358 | PX425498 |
| JAUCC1853 | Jiangxi Province | PX376359 | PX425499 |
| JAUCC1854 | Jiangxi Province | PX376360 | PX425500 |
| JAUCC1857 | Jiangxi Province | PX376361 | PX425501 |
| JAUCC1858 | Jiangxi Province | PX376362 | PX425502 |
| JAUCC1859 | Jiangxi Province | PX376363 | PX425503 |
| JAUCC1860 | Jiangxi Province | PX376364 | PX425504 |
| JAUCC1861 | Jiangxi Province | PX376365 | PX425505 |
| JAUCC1862 | Jiangxi Province | PX376366 | PX425506 |
| JAUCC1913 | Fujian Province | PX376367 | PX425507 |
| JAUCC1914 | Fujian Province | PX376368 | PX425508 |
| JAUCC1915 | Fujian Province | - | PX425509 |
| JAUCC1920 | Yunnan Province | PX376369 | PX425510 |
| JAUCC1921 | Yunnan Province | PX376370 | PX425511 |
| JAUCC1922 | Yunnan Province | PX376371 | PX425512 |
| JAUCC1925 | Yunnan Province | - | PX425513 |
| JAUCC1926 | Yunnan Province | PX376372 | PX425514 |
| JAUCC1927 | Yunnan Province | PX376373 | PX425515 |
| JAUCC2107 | Shandong Province | PX376374 | PX425516 |
| JAUCC2108 | Shandong Province | PX376375 | PX425517 |
| JAUCC2109 | Jiangsu Province | - | PX425518 |
| JAUCC2113 | Beijing | PX376376 | PX425519 |
| JAUCC2114 | Zhejiang Province | - | PX425520 |
| JAUCC2115 | Zhejiang Province | PX376377 | PX425521 |
| JAUCC2116 | Shandong Province | PX376378 | PX425522 |
| JAUCC2117 | Heilongjiang Province | PX376379 | PX425523 |
| JAUCC2118 | Hubei Province | PX376380 | PX425524 |
| JAUCC2119 | Hubei Province | PX376381 | PX425525 |
| JAUCC2120 | Sichuan Province | PX376382 | PX425526 |
| JAUCC2121 | Sichuan Province | PX376383 | PX425527 |
| JAUCC2122 | Sichuan Province | PX376384 | PX425528 |
| JAUCC2123 | Sichuan Province | PX376385 | PX425529 |
| JAUCC2124 | Sichuan Province | PX376386 | PX425530 |
| JAUCC2125 | Sichuan Province | PX376387 | PX425531 |
| JAUCC2126 | Sichuan Province | PX376388 | PX425532 |
| JAUCC2127 | Sichuan Province | PX376389 | PX425533 |
| JAUCC2128 | Sichuan Province | PX376390 | PX425534 |
| JAUCC2180 | Guangdong Province | PX376391 | PX425535 |
| JAUCC2181 | Guangdong Province | PX376392 | PX425536 |
| JAUCC2182 | Shanghai | PX376393 | PX425537 |
| JAUCC2183 | Shandong Province | PX376394 | PX425538 |
| JAUCC2184 | Hubei Province | PX376395 | PX425539 |
| JAUCC2185 | Hubei Province | PX376396 | PX425540 |
| JAUCC2187 | Shandong Province | - | PX425541 |
| JAUCC2188 | Zhejiang Province | PX376397 | PX425542 |
| JAUCC2189 | Jiangsu Province | PX376398 | PX425543 |
| JAUCC2190 | Shanghai | - | PX425544 |
| JAUCC2191 | Shandong Province | - | PX425545 |
| JAUCC2192 | Shandong Province | PX376399 | PX425546 |
| JAUCC2193 | Shandong Province | PX376400 | PX425547 |
| JAUCC2194 | Sichuan Province | - | PX425548 |
| JAUCC2195 | Shandong Province | PX376401 | PX425549 |
| JAUCC2196 | Shandong Province | PX376402 | PX425550 |
| JAUCC2197 | Sichuan Province | PX376403 | PX425551 |
| JAUCC2198 | Hubei Province | PX376404 | PX425552 |
| JAUCC2199 | Hubei Province | PX376405 | PX425553 |
| JAUCC2200 | Shandong Province | PX376406 | PX425554 |
| JAUCC2201 | Shandong Province | - | PX425555 |
| JAUCC2202 | Liaoning Province | PX376407 | PX425556 |
| JAUCC2203 | Liaoning Province | PX376408 | PX425557 |
| JAUCC2205 | Guangdong Province | PX376409 | PX425558 |
| JAUCC2206 | Sichuan Province | PX376410 | PX425559 |
| JAUCC2207 | Guangdong Province | PX376411 | PX425560 |
| JAUCC2211 | Hubei Province | PX376412 | PX425561 |
| JAUCC2212 | Hubei Province | PX376413 | PX425562 |
| JAUCC2213 | Jilin Province | PX376414 | PX425563 |
| JAUCC2214 | Jilin Province | PX376415 | PX425564 |
| JAUCC2218 | Jiangxi Province | PX376416 | PX425565 |
| JAUCC2223 | Fujian Province | PX376417 | PX425566 |
| JAUCC2330 | Shandong Province | PX376418 | PX425567 |
| JAUCC2332 | Shandong Province | PX376419 | PX425568 |
| JAUCC2350 | Jiangsu Province | PX376420 | PX425569 |
| JAUCC2924 | Yunnan Province | PX376421 | PX425570 |
| JAUCC2925 | Yunnan Province | PX376422 | PX425571 |
| JAUCC4926 | Jiangxi Province | PX376423 | PX425572 |
| JAUCC5422 | Jiangxi Province | PX376424 | PX425573 |
| JAUCC5626 | Jiangxi Province | PX376425 | PX425574 |
| JAUCC5627 | Jiangxi Province | PX376426 | PX425575 |
| JAUCC5628 | Jiangxi Province | PX376427 | PX425576 |
| JAUCC5629 | Jiangxi Province | PX376428 | PX425577 |
| JAUCC5630 | Beijing | PX376429 | PX425578 |
| JAUCC5631 | Beijing | PX376430 | PX425579 |
| JAUCC5632 | Sichuan Province | PX376431 | PX425580 |
| JAUCC5633 | Jiangxi Province | PX376432 | PX425581 |
| JAUCC5634 | Guangdong Province | PX376433 | PX425582 |
| JAUCC5635 | Jiangsu Province | PX376434 | PX425583 |
| JAUCC5636 | Beijing | PX376435 | PX425584 |
| JAUCC5637 | Beijing | PX376436 | PX425585 |
| JAUCC5638 | Beijing | PX376437 | PX425586 |
